# Optically Transparent Tri-Wideband Mosaic Frequency Selective Surface with Low Cross-Polarisation

**DOI:** 10.3390/ma15020622

**Published:** 2022-01-14

**Authors:** Nur Biha Mohamed Nafis, Mohamed Himdi, Mohamad Kamal A Rahim, Osman Ayop, Raimi Dewan

**Affiliations:** 1 School of Electrical Engineering, Faculty of Engineering, Universiti Teknologi Malaysia, Johor Bahru 81310, Malaysia; bihanafis@yahoo.com (N.B.M.N.); mdkamal@utm.my (M.K.A.R.); osmanayop@utm.my (O.A.); 2 Department of Physics, Faculty of Science, Universiti Putra Malaysia, Serdang, Selangor 43400, Malaysia; 3 Institut d’Electronique et des Technologies du numéRique (IETR), UMR CNRS 6164, Université de Rennes 1, Campus de Beaulieu, 35042 Rennes, France; 4 Advanced Diagnostics and Progressive Human Care, School of Biomedical Engineering and Health Sciences, Faculty of Engineering, Universiti Teknologi Malaysia, Johor Bahru 81310, Malaysia; raimi.dar@utm.my

**Keywords:** mosaic frequency selective surface, fractal, optical transparency, wideband, low cross-polarisation

## Abstract

Acquiring an optically transparent feature on the wideband frequency selective surface (FSS), particularly for smart city applications (building window and transportation services) and vehicle windows, is a challenging task. Hence, this study assessed the performance of optically transparent mosaic frequency selective surfaces (MFSS) with a conductive metallic element unit cell that integrated Koch fractal and double hexagonal loop fabricated on a polycarbonate substrate. The opaque and transparent features of the MFSS were studied. While the study on opaque MFSS revealed the advantage of having wideband responses, the study on transparent MFSS was performed to determine the optical transparency application with wideband feature. To comprehend the MFSS design, the evolutionary influence of the unit cell on the performance of MFSS was investigated and discussed thoroughly in this paper. Both the opaque and transparent MFSS yielded wideband bandstop and bandpass responses with low cross-polarisation (−37 dB), whereas the angular stability was limited to only 25°. The transparent MFSS displayed high-level transparency exceeding 70%. Both the simulated and measured performance comparison exhibited good correlation for both opaque and transparent MFSS. The proposed transparent MFSS with wideband frequency response and low cross-polarisation features signified a promising filtering potential in multiple applications.

## 1. Introduction

Future interest in the rapid evolution of 5G technology and the development of smart cities requires antennas [1,2,3] and frequency selective surfaces (FSS) to be optically transparent when integrated into the existing infrastructures [4]. This integration fosters sustainable 5G wireless network needs and microwave filtering for additional security. The FSS structure is a periodic array that permits the transmission or blocking of the electromagnetic (EM) wave signal based on the array geometries, namely patch and slot. The incorporation of array geometries (patch and slot) yield the desired frequency response, such as the bandpass and bandstop [5,6]. Hence, FSS has been widely used in a variety of applications, including EM shielding [7], shipboard radar [8], and radome [9]. However, single-layer FSS is always associated with narrowband operation [10,11,12]. Although the implementation of a fractal structure onto FSS allows multiband frequency response that is insensitive towards angular variation, it has narrow bandwidth (BW) [13,14,15,16]. Meanwhile, multilayer FSS can yield promising results for BW enhancement [5,17,18,19] and is insensitive to angular variation [20,21,22], which in turn increases the implementation cost along with the end product’s size and thickness [23]. The literature [8,24,25,26,27] depicts that wideband frequency response may also be achieved using single layer FSS–FSS with a single layer metallic conductor on a single layer substrate configuration. For instance, the modification of a square loop by adding narrow metallic strips at the four corners of the loop enhances the BW [24]. Redistribution of the current along the strips reduces the surface current path length, thus enhancing the BW without altering the size dimension of the unit cell. Nonetheless, due to the wide trace width of the structural element, the previously proposed FSS could not be applied for optical transparency applications.

Hence, research on optically transparent single layer FSS has become an interesting topic to be explored lately [28,29,30,31,32,33]. Initially, thin-film technique with transparent conductive material, such as indium tin oxide (ITO), silver-coated polyester film (AgHT), and fluorine-doped tin oxide (FTO), were commonly used to obtain high optical transparency for both antenna and FSS. However, the limited availability of ITO has led to a surge in its price [34]. Furthermore, many issues, including the imperfect continuity of periodic array fabrication, lack of flexibility, and easy deterioration as well as mechanical, thermal, and chemical stability issues of ITO, were identified from the use of ITO [34,35]. Moreover, the air-gap between the thin film (AgHT) and the substrate used, due to the adhesive material used in between, increases the thickness of the prototype and affects the prototype performance [36].

Meanwhile, the meshed technique initiated by [37] is an alternative to the classical thin-film technique for optical transparency application. The meshed metal film is made up of conductor, such as copper or silver, with appropriate grid pattern within the design topology. The meshed technique has been explored widely for both passive [37,38] and active [39] transparent antenna applications, and currently expanded for transparent FSS applications that are insensitive towards angular and polarised variations [32,40]. However, mesh grid size and mesh line width are the two factors that must be considered due to their impact on both the performance and the optical transparency of FSS [40]. When using the meshed technique, the frequency response of FSS is not only limited to single band FSS, but triple band FSS can also be developed by using the meshed technique [32]. Recently, electrotextiles (or metal–mesh fabrics) composed of copper microgrids (e.g., RadioScreen and VeilShield) placed on acrylic substrate have been applied for transparent FSS due to several factors, including low in cost, ease of fabrication process, highly transparent, and waterproof features—suitable for 5G applications [33]. In fact, more than 50% and 70% of optical transparency can be achieved by using RadioScreen and VeilShield, respectively. Nevertheless, the thickness of the electrotextiles is a concern in FSS degraded performance, especially during the fabrication process. To date, only transparent FSS with narrow BW is achievable by using this technique.

At present, the screen-printing technique is favourable for FSS fabrication. Screen-printing for flexible FSS [30,41,42], security paper FSS [43,44], absorber-based FSS [45], and millimetre-wave radar absorbing materials based FSS [46] are among the explored applications concerning the use of screen-printing technique for FSS fabrication. Apart from being cost effective, this technique is a relatively simple fabrication procedure, especially for large periodic array that is suitable for mass production in industry [30]. Even for a complex and miniature-size dimension of the unit cell, this technique provides high precision and resolution for FSS fabrication [42]. As for the optical transparency features of FSS, the exploration of the use of screen-printing technique is still rarely documented. The screen-printing technique has been used to fabricate transparent FSS [30]. However, due to the wide trace width of the structural element, only semi-transparent FSS can be achieved by using this technique.

Therefore, this study proposes a high optically transparent single layer mosaic frequency selective surface (MFSS) with wideband bandstop and bandpass frequency responses, as well as low cross-polarisation. Transparent MFSS is made up of a conductive metallic element unit cell combining Koch fractal and double hexagonal loop on a polycarbonate substrate. This paper is structured into five sections. Section 2 describes the proposed MFSS design in detail, followed by the simulation results of MFSS unit cell evolution and the optical transparency of the MFSS in Section 3. Section 4 discusses the fabrication and measurement process of the MFSS. Finally, Section 5 concludes and summarises the study.

## 2. MFSS

**Figure 1:**
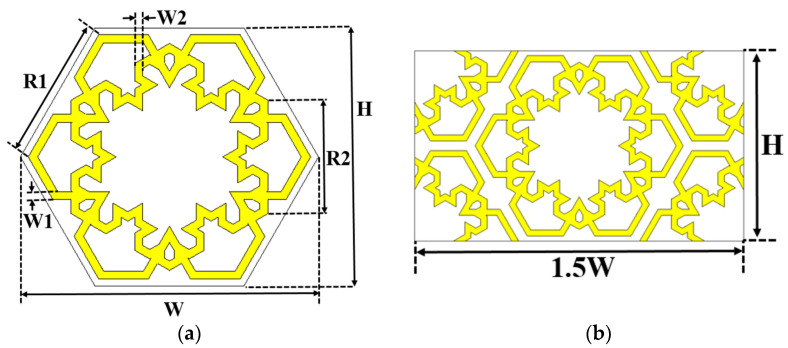
The geometry of the MFSS. (**a**) Unit cell of the MFSS, and (**b**) triangular lattice arrangement. (H = 7.01 mm, P = 8.09 mm, R1 = 3.84 mm, R2 = 3.08 mm and W1 = W2 = 0.22 mm).

In this study, the concept described in [24] was explored by applying interconnection between the double hexagonal loop structures to determine the possibility of having wideband response. The BW of the bandstop frequency response should generate a wider BW, instead of being selective with narrow BW of the basic loop structure. As for the MFSS, fractal structure (e.g., Koch fractal) was selected due to its ability to increase the side length of the unit cell without affecting the initial unit cell size dimension. Figure 1a illustrates the proposed unit cell structure of MFSS that combines the first and second iteration level of Koch fractal on the outer and the inner loops of the double hexagonal loop, respectively. The inner loop is positioned at a rotation of 30°, allowing the interconnection between both loops for enhancement of BW. Figure 1b depicts the unit cells’ triangular lattice arrangement that minimises gap spacing for BW enhancement, yields stable frequency response performance and avoids the grating lobe phenomena [47]. The rectangular pattern of the MFSS (1.5 width (W) × height (H)) was used for simulation purposes.

The advantage of MFSS with wideband bandpass and bandstop responses was identified based on opaque MFSS. In particular, this study assessed the use of MFSS for optically transparent application with wideband feature—transparent MFSS. Two MFSSs with identical parameters of the proposed FSS unit cell were assessed in this study:

(1) An opaque MFSS as a reference, fabricated on an FR4 substrate with a permittivity ($\varepsilon_{r})$ of 4.3, loss tangent (tandδ) of 0.02, lossy substrate thickness of 1.6 mm and copper layer thickness of 0.035 mm with a conductivity (σ) of $5.8\times 10^{7}$ S/m.
(2) A transparent MFSS using a polycarbonate substrate with a $\varepsilon_{r}$ of 2.9, tandδ of 0.005, substrate thickness of 1.5 mm and a silver layer thickness of 0.006 mm with a conductivity (σ) of $4.3\times 10^{6}$ S/m.

## 3. Simulation Results

The frequency response simulation of the MFSS was performed using Computer Simulation Technology (CST) Microwave Studio software.

### 3.1. Wideband MFSS—Evolution of Unit Cell (Opaque)

**Figure 2:**
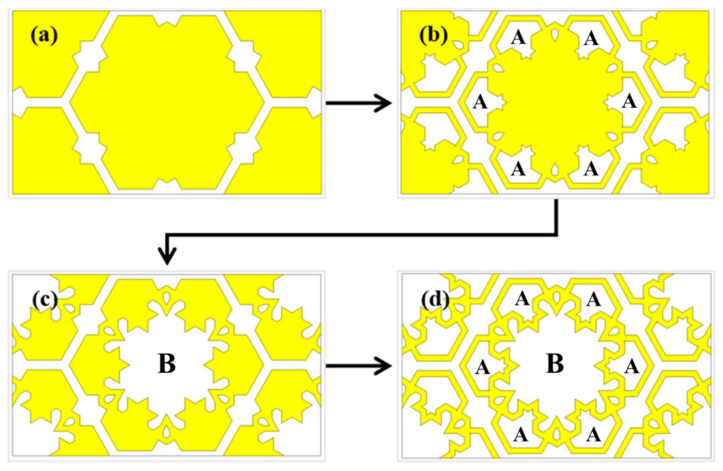
Unit cell evolution of the proposed FSS; (**a**) FSS 1, (**b**) FSS 2, (**c**) FSS 3, and (**d**) MFSS.

This section discusses the evolution of the unit cell to comprehend the geometrical structure of the proposed MFSS, which used FR4 as the substrate for all unit cells. The reverse analysis was conducted using the proposed MFSS to understand the changes in the frequency response for every structural transformation. According to Figure 2, a hexagonal patch (FSS 1) (see Figure 2a) was initiated by having patches on all the uncovered areas of the MFSS unit cell, including parts A and B of the proposed MFSS. The analysis proceeded by introducing patches in part B of the proposed MFSS and produced six small loops in part A of the proposed MFSS (FSS 2) as illustrated in Figure 2b. In the unit cell depicted in Figure 2c, part A of the proposed MFSS unit cell was covered with patches to form a single big loop (FSS 3). Figure 2d demonstrates the proposed MFSS with no patches in parts A and B of the unit cell.

**Figure 3:**
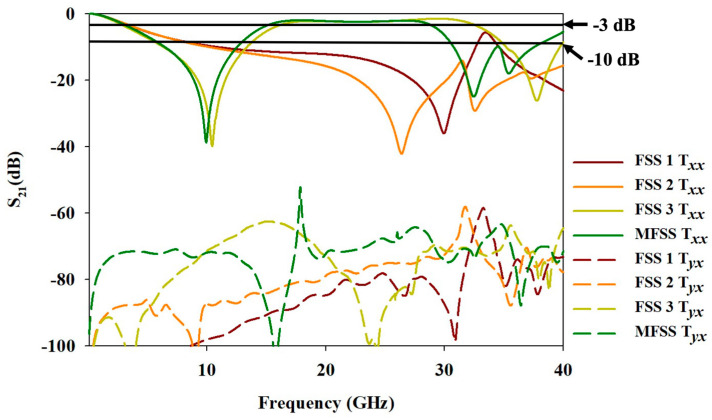
The simulated $S_{21}$ from the evolution of MFSS unit cell.

Figure 3 illustrates the simulated transmission coefficient ($S_{21}$) of the evolution of MFSS unit cell (FSS 1, FSS 2, FSS 3, and MFSS) under co-polarisation ($T_{xx}$) and cross-polarisation ($T_{yx}$) incidences. The BW reference level depends on the frequency responses of FSS which include $S_{21}\leq$ −10 dB for bandstop frequency response and $S_{21}\geq$ −3 dB for bandpass frequency responses.

Based on Figure 3, the simulated $T_{xx}$ frequency response for FSS 1 exhibited a nearly complete metal behaviour, as the incident wave was completely blocked by reflecting all EM waves over an extensive range of frequency. The BW ranged from 10.69 GHz to 32.62 GHz (BW of 21.93 GHz) with a fractional bandwidth (FBW) of 101.27% and a resonant frequency ($f_{r}$) of 29.95 GHz.

The FSS 2 also exhibited a similar trend of simulated $T_{xx}$ frequency response to that of FSS 1 as depicted in Figure 3. The $f_{r}$ shifted to a lower frequency region of 26.4 GHz with the addition of a secondary $f_{r}$ resonated at 32.54 GHz, allowing a broader BW noted at 29.89 GHz (FBW of 119.30%) within the range of 10.11 GHz to 40 GHz. The EM wave signal lower than 10.11 GHz was permitted to transmit through the FSS 2, while the EM wave above 10.11 GHz was blocked.

When the unit cell evolved to FSS 3, the simulated $T_{xx}$ frequency response exhibited bandstop responses at low- and high-frequency bands with a bandpass frequency response, which is generally portrayed by the combination of the patch and slot geometry [6]. The first bandstop frequency response of FSS 3 resonated at $f_{r}$ of 10.43 GHz with a BW of 7.01 GHz (FBW of 71.42%), starting from 6.31 GHz to 13.32 GHz. The bandpass frequency response recorded a BW of 15.63 GHz (within 16.54 GHz to 32.17 GHz), with an FBW of 64.18%. Whereas, the EM wave was prohibited from passing through the FSS 3 beyond 35.13 GHz to 39.67 GHz (FBW of 12.14%), with a frequency resonating at 37.73 GHz. The frequency response of the FSS 3 significantly changed from a low pass frequency response to a combination of bandstop and bandpass frequency response with a high FBW by having patches at part A of the proposed MFSS.

Finally, the first bandstop of the simulated $T_{xx}$ frequency response for the MFSS resonated at a $f_{r}$ of 9.94 GHz with a BW of 6.10 GHz (FBW of 64.35%), within a frequency range of 6.43 GHz to 12.53 GHz. Together, the bandpass frequency response was within a frequency range of 15.49 GHz to 28.53 GHz, with a BW of 13.04 GHz and FBW of 59.25%. Meanwhile, the second bandstop frequency response resonated at 32.46 GHz with a secondary $f_{r}$ at 35.41 GHz, with a BW of 6.81 GHz (FBW of 19.94%), which lied within a frequency range of 30.74 GHz to 37.55 GHz.

**Figure 4:**
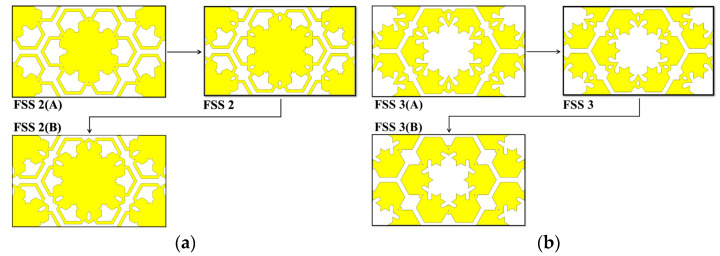
The structural changes of (**a**) FSS 2 and (**b**) FSS 3.

**Figure 5:**
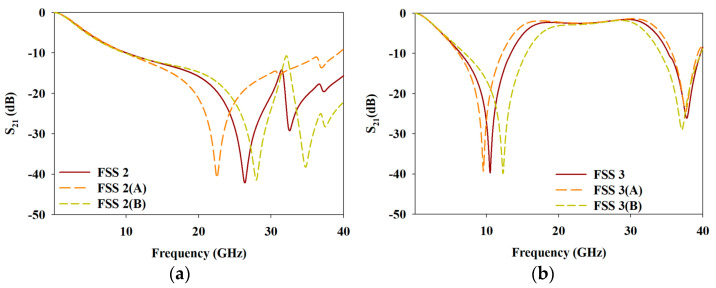
The frequency response under the influence of structural changes of (**a**) FSS 2 and (**b**) FSS 3.

To better understand the effects of the structural changes (FSS 2 and FSS 3) on frequency response, Figure 4 illustrates the changes in aperture area of parts A and B for FSS 2 and FSS 3, respectively. Meanwhile, Figure 5 depicts the frequency response under the influence of structural changes of FSS 2 and FSS 3. According to Figure 4a, FSS 2(A) has a larger aperture area compared to FSS 2(B) with a smaller aperture area of part A than that of FSS 2. Based on Figure 5a, the same low pass frequency response was achieved with the structural changes of FSS 2. However, the $f_{r}$ of the FSS 2(A) shifted towards a lower frequency region while the $f_{r}$ of FSS 2(B) shifted to higher frequency region with enhanced BW of the FSS 2(B), greater than 40 GHz.

On the other hand, Figure 4b compares FSS 3(A) and FSS 3(B) with the initial FSS 3. Based on the observation, FSS 3(A) yielded a larger aperture area compared to the smaller aperture area of FSS 3(B) of part B. According to Figure 5b, FSS 3(A) yielded a narrower first bandstop frequency response with a shift to a lower frequency region of $f_{r}$, while the BW of the bandpass frequency response widened. The second bandstop frequency response remained nearly constant. As for FSS 3(B), the $f_{r}$ of the first bandstop frequency response shifted to higher frequency region where the BW was further enhanced. The bandpass frequency response with narrower BW was achieved, while the second bandstop frequency response experienced a slight shift of the $f_{r}$ to lower frequency region, with a slightly wider BW.

Based on the aperture area comparison between FSS 2 and FSS 3, the aperture of part A of FSS 2 enhanced the BW of the bandstop frequency response, especially at a higher frequency region. Whereas, the aperture of part B of FSS 3 significantly affected the first bandstop and the bandpass frequency responses, but was less impactful on the second bandstop frequency response. The introduction of aperture in part A of FSS 2 to FSS 3 to yield proposed MFSS, resulted in an enhanced BW in the second bandstop frequency response by ~ 8% from 12% for FSS 3 to 20% for MFSS. Despite a slight decrease in FBW and a slight shift of $f_{r}$ when the proposed MFSS was compared with FSS 3, the frequency responses at the first bandstop and bandpass frequency responses were still considered to be a wideband as FBW achieved above 50%.

The simulated $T_{yx}$ values for the frequency range, especially at the bandpass frequency response, differed vastly from the $T_{xx}$ values. Figure 3 shows that the $T_{yx}$ values of the evolution of MFSS unit cell were lower than the approximation of −52 dB for the frequency range, especially within the bandpass, signifying a good reduction of cross-polarisation with use of opaque MFSS.

**Figure 6:**
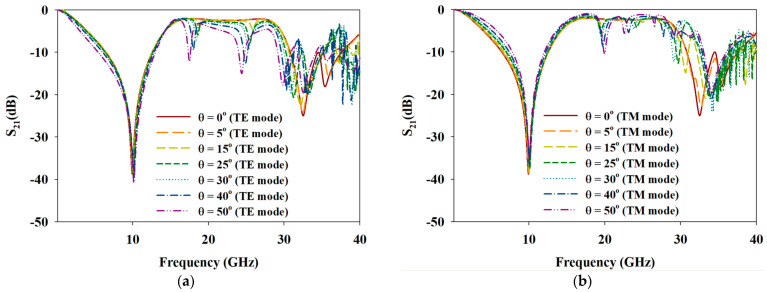
Simulated responses of opaque MFSS under the influence of oblique angle variations of (**a**) TE mode and (**b**) TM mode incidents.

The frequency responses of the opaque MFSS under the influence of oblique angle variations (0°–50°) for TE and TM mode incidences are shown in Figure 6a,b, respectively. Under both TE and TM modes with varied oblique angles, the opaque MFSS exhibited near similar responses, especially at the first bandstop frequency response. However, degradation of BW was noted for bandpass and the second bandstop frequency responses as the oblique angles increased above 10°. Among the reasons that limit the performance of angular stability are the grating lobes phenomenon and Wood’s Anomalies phenomenon (scan blindness phenomenon). The grating lobes phenomenon occurs when the gap spacing between adjacent unit cells or the periodicity of the unit cell is electrically large, which eventually deteriorates the performance of the FSS when the EM wave hits the surface of FSS from different angles [48]. As for the proposed MFSS, the lattice type used is a triangular spacing, in which the general rule applied is that the gap spacing between adjacent unit cells must be less than 1.15 $\lambda_{o}$ for oblique angle that less than 45°, and 0.67 $\lambda_{o}$ for oblique angle that 45° and above [47]. The periodicity of the unit cell for the proposed MFSS was 0.23 $\lambda_{o}$. The frequency responses of the proposed MFSS for both TE and TM modes were predicted to remain unchanged as the oblique angles increased from 0° to 50°. However, according to Figure 6, the Wood’s Anomalies phenomenon also known as the scan blindness phenomenon [48,49,50] was observed when the oblique angle increased up to 50°, especially for frequency ranging higher than 15 GHz (TE mode) and 19 GHz (TM mode).

Wood’s Anomalies phenomenon can be defined as “rapid variations in the intensity of the various diffracted spectral orders in certain narrow frequency bands” [48]. It commonly occurs at a frequency region similar to or higher than the resonant frequency. 

As mentioned in [50], the three main reasons that contributed to the scan blindness phenomenon include:

(1) The propagation constant β equals a surface wave propagation constant $\beta_{SW}$.
(2) The grid spacings a, b are such that the equality of propagation constants in (1) occurs for values of u, v in real space.
(3) The TM (TE) surface wave pole of (1) is not cancelled by a zero value of $k_{x}$($k_{y}$).

The scan blindness occurs when a portion of a surface wave circle intersects the visible space region $\left({\left|u\right|}^{2}+{\left|v\right|}^{2}<1\right)$, which satisfies the contribution of (1) and (2). However, the occurrence of polarisation mismatch could cause the particular surface wave pole to be cancelled.

The triangular lattice arrangement of the MFSS unit cell was chosen due to its ability to minimise the periodicity of the unit cell to avoid the grating lobe phenomenon. However, the triangular lattice arrangement has no advantage to avoid the scan blindness phenomenon [50]. Based on the proposed MFSS, one of the reasons that contributed to this phenomenon is the substrate’s thickness and permittivity. The increased substrate’s permittivity and thickness shift the $f_{r}$ to a lower frequency region. According to [50], for example, with the λ/2 of gap spacing between adjacent unit cells, scan blindness will occur because the propagation constant $\left(\beta_{SW}/k_{o}\right)$ is greater than 1. For a lower substrate permittivity, multiple scan blindness would occur when substrate’s thickness is as thick as 0.5 λ. For higher substrate permittivity, multiple surface wave circles are presence in visible space when the substrate’s thickness is only as thin as 0.3 λ. It subsequently leads to multiple regions of scan blindness instead of thick substrate with lower permittivity. 

To mitigate the scan blindness phenomenon in the proposed MFSS, grid spacing between adjacent elements, along with the substrate’s thickness and permittivity must be adequately considered. The grid spacing should be less than λ/2, with low substrate permittivity and thin substrate to prevent the surface wave circles from entering the visible space. Since the available FR4 in the market is thick and possesses high permittivity, scan blindness phenomenon is expected to be presence, especially at higher frequency range. 

### 3.2. Surface Current Distribution

This section discusses the surface current distribution of the unit cell evolution for MFSS at resonated frequencies, in order to better understand the resonance mechanism of MFSS. The surface current distribution was attained as a result of the excitation of normal incident EM plane wave under TE polarisation. The direction of the surface current distribution is indicated by the arrow, whereas the magnitude of the surface current is indicated by colour.

**Figure 7:**
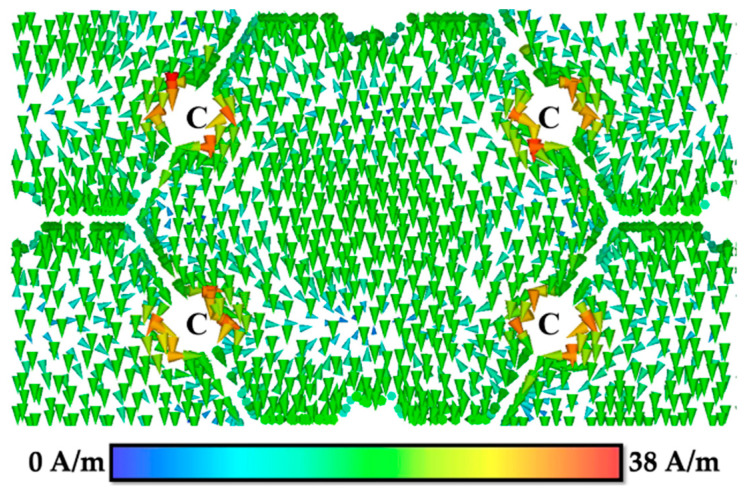
Surface current distribution of FSS 1 at 29.95 GHz.

Referring to Figure 7, at $f_{r}$ = 29.95 GHz, the surface current distribution on FSS 1 was flow in negative *y*-direction and the current was entirely weak through the conductor surface, except for the center edge of the four sides of FSS 1 (C part).

**Figure 8:**
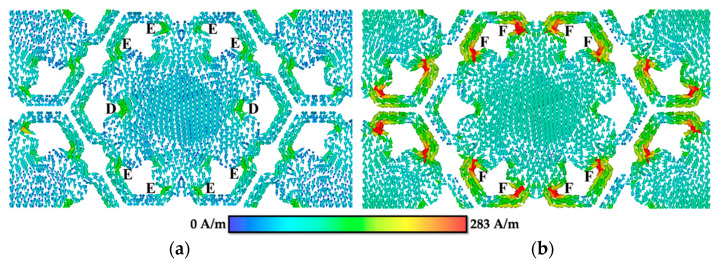
Surface current distribution of FSS 2 at (**a**) 26.4 GHz and (**b**) 32.54 GHz.

In the surface current distribution for FSS 2 (see Figure 8a) at $f_{r}$ = 26.4 GHz, the surface current mainly concentrated at the edge sides of the centre patch (D part), as well as at the area between patch integration and small loops (E part), which flowed in negative *y*-direction. For surface current distribution at $f_{r}$ = 32.54 GHz (see Figure 8b), the surface current flowed in positive *y*-direction and concentrated at the four loops situated at the top and bottom of FSS 2 (F part). At each loop, two current paths were formed with the surface current flowing in opposite direction, thus creating a parallel loop [24], forming a magnetic dipole. The surface current distribution radiates strongly to the free space, allowing a broader resonance in the transmission spectrum to be achieved [51].

**Figure 9:**
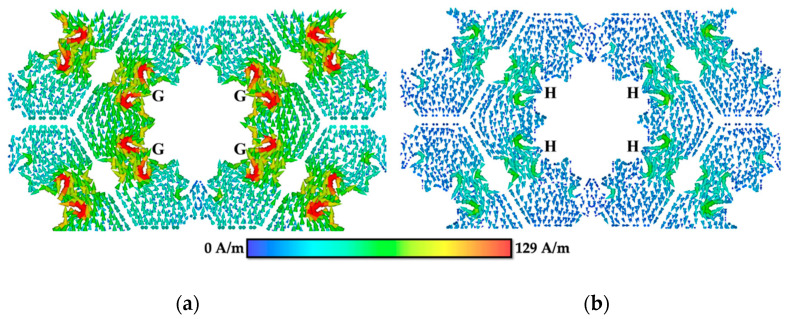
Surface current distribution of FSS 3 at (**a**) 10.43 GHz and (**b**) 37.73 GHz.

For FSS 3, introducing patches at part the A of the MFSS yielded a single big loop, as illustrated in Figure 9. The generated parallel loop of the surface current distribution flowed in positive *y*-direction. Figure 9a shows that at $f_{r}$ = 10.43 GHz, the surface current distribution was stronger at the edge centre inner sides of the current paths (G part), but weak at the two ends of the paths that equally divided the path length, which was situated at the centre of the top and bottom parts of FSS 3. This was similar for surface current distribution at $f_{r}$ = 37.73 GHz (see Figure 9b). The surface current of the parallel loop flowed in negative *y*-direction and concentrated at the centre edge of the four sides of FSS 3 (H part). Similar to FSS 2, the parallel loop of the surface current distribution formed the magnetic dipole. As for FSS 3, a broader resonance was obtained at both $f_{r}$ in the transmission spectrum.

**Figure 10:**
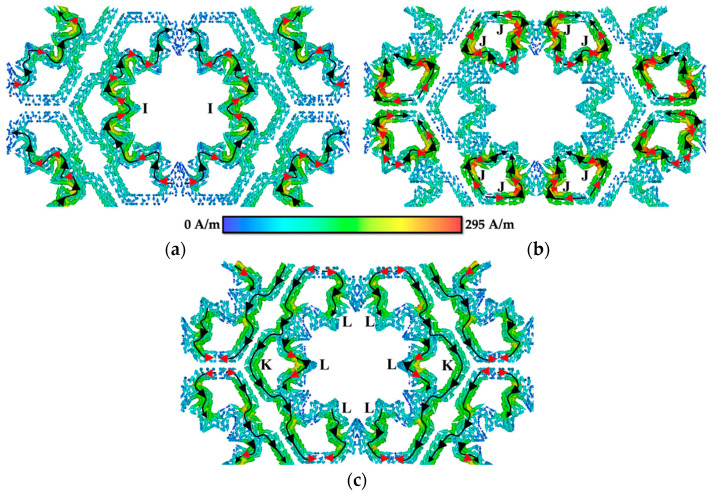
Surface current distribution of opaque MFSS at (**a**) 9.94 GHz, (**b**) 32.46 GHz, and (**c**) 35.41 GHz.

Figure 10 presents the surface current distribution of MFSS, where no patches were introduced to parts A and B of the MFSS, generating a big inner loop with six surrounding small loops. At $f_{r}$ = 9.94 GHz, the surface current of the parallel loop flowed in positive *y*-direction and concentrated at the edge center inner sides of the inner big loop current paths (I part), forming a magnetic dipole, as shown in Figure 10a. By comparing FSS 3 with MFSS, $f_{r}$ at lower frequency range was obtained mainly due to inner aperture (B part), as in this case, the single inner loop of MFSS (I part). 

For the surface current distribution at $f_{r}$ = 32.46 GHz shown in Figure 10b, strong surface current was obtained at the four parallel loops (J part) of small loops, situated at the top and bottom of the MFSS, thus flowing in the positive *y*-direction. By comparing the surface current distribution of FSS 2 and MFSS, it can be deduced that the six small loops that were created due to the introduction of the patches in part B of the MFSS contributed to the primary $f_{r}$ at higher frequency region.

The surface current distribution of the additional secondary $f_{r}$ = 35.41 GHz is illustrated in Figure 10c. Two parallel loops were observed, in which the strong surface current was attained at both the six small outer loops (K part) and the inner (L part) parts of the MFSS, and flowed in negative *y*-direction. Referring to Figure 10c, the current paths yielded two parallel loops (formation of a magnetic dipole), at the right and left sides of the MFSS, and the surface current mainly concentrated along the outer loops (K part) and at certain parts of the inner loop (L part). Hence, it can be concluded that the additional secondary $f_{r}$ at high frequency region was generated due to the integration between the inner and outer loops of the MFSS, thus allowing a wider FBW of 19.94% to be achieved at higher frequency region, instead of only 12.14% for FSS 3.

Figure 10 illustrates the physical mechanism of cross-polarisation. Based on Figure 10a–c, the black arrows indicate the flow of the surface current distribution in a positive *y*-direction, while the red arrow refers to the surface current distribution in both the positive and negative *x*-directions. The magnetic field generated for the surface current distribution flowed in the positive *x*-direction was along the negative *y*-direction. While the magnetic field for the surface current flowed in the negative *x*-direction was generated in the opposite direction – positive *y*-direction. It can be observed that the induced electric field at the *x*-direction (red arrow) was perpendicular to the incident electric field, and in the same direction of the incident magnetic field, thus a polarisation deflection was not produced. Moreover, since the induced magnetic field in the *y*-direction was parallel to the incident electric field, a 90° polarisation deflection was produced. The magnetic field of the opposite direction generated by the horizontal surface current distribution cancelled each other, led to low cross-polarisation and high isolation from the co-polarisation value.

### 3.3. Optical Transparency of MFSS

In realising the optically transparent application of the MFSS, the periodic array of MFSS was fabricated on a transparent polycarbonate substrate, as described in Section 2. A transparent FSS should at least have 50% optical transparency to be qualified as a transparent FSS [52]. The optical transparency (*T*) of the evolution of MFSS unit cell (FSS 1, FSS 2, FSS 3, and MFSS) was estimated using Equation (1) [4] (see Table 1). Table 1 compares the optical transparency of MFSS with the rest of the MFSS unit cell evolution. Due to the complexity of the unit cell, the total area (*TA*) and the metal element area (*MA*) of the unit cell was obtained using CST software.

(1) $$T\left(\%\right)=\frac{TA-MA}{TA}$$

**Figure 11:**
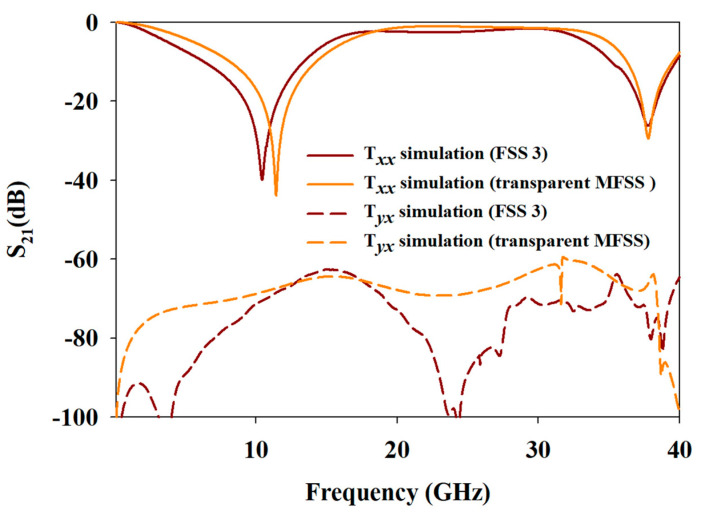
The simulated T*_xx_* and T*_yx_* of FSS 3 and transparent MFSS.

The computed $T_{xx}$ and $T_{yx}$ of opaque FSS 3 and transparent MFSS are illustrated in Figure 11. The first bandstop frequency response of the transparent MFSS provided signal attenuation up to 44 dB at 11.43 GHz, with BW of 6.07 GHz and FBW of 54.22%, within the frequency range of 8.16–14.23 GHz. As for the bandpass frequency response, the transparent MFSS allowed the transmission of the incident EM wave from 17.67 GHz to 33.84 GHz with a BW of 16.17 GHz and FBW of 62.78%. When compared with the first bandstop and bandpass frequency responses of FSS 3 with FBW of 71.42% and 64.18% respectively, despite the slight decrease in FBW and a slight shift of $f_{r}$ from 10.43 GHz, the responses of the transparent MFSS were still considered as wideband as the FBW achieved above 50%.

The $f_{r}$ resonated at 37.77 GHz for the second bandstop frequency response, with BW of 3.23 GHz and FBW of 8.53%, within the frequency ranging at 36.27–39.50 GHz. When compared with the FSS 3 with FBW of 12.14% and $f_{r}$ maintained at 37.73 GHz, the FBW decreased by 4% and the $f_{r}$ remained nearly unchanged. The deviation in BW stemmed from substrate permittivity of the transparent MFSS.

Referring to Table 1 and based on the above discussion, it can be concluded that near similar bandstop and bandpass attributes of opaque FSS 3 can be achieved by using the transparent MFSS, with the perks of having high optical transparency of 70.3%, instead of only 42.0% for FSS 3.

Based on the simulated $T_{yx}$ of transparent MFSS shown in Figure 11, the $T_{yx}$ values were below −60 dB for all frequency responses, especially within the bandpass frequency region.

**Figure 12:**
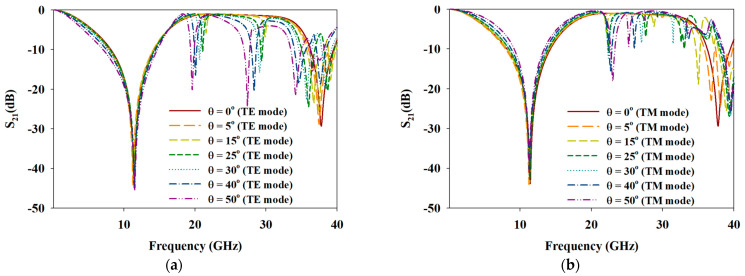
Simulated responses of transparent MFSS under the influence of oblique angle variations of (**a**) TE mode and (**b**) TM mode incidents.

Figure 12a,b illustrates the frequency responses of transparent MFSS under the influence of oblique angle variation for TE and TM mode incidences. Notably, the frequency responses for transparent MFSS, especially for the first bandstop frequency response, displayed near similar response as the oblique angles that increased from 0° to 50°. However, for the bandpass frequency response, two resonated frequencies were observed at ~ 20 GHz and 30 GHz with very narrow BW, which became more apparent as the oblique angle increased. For the second bandstop frequency response, the degradation of BW and the shift of $f_{r}$ were observed when the oblique angle exceeded 15°. As mentioned in Section 3.1, the grating lobe phenomenon and Wood’s Anomalies phenomenon (scan blindness phenomenon) are among the reasons that limit the FSS performance in angular stability. According to Figure 12, the scan blindness phenomenon can be easily observed as the oblique angle increased up to 50°, especially for high frequencies ranging greater than 18 GHz (TE mode) and 20 GHz (TM mode), possibly due to the substrate’s permittivity and thickness. A thinner polycarbonate substrate is expected to mitigate the scan blindness phenomenon.

## 4. Experiment

**Figure 13:**
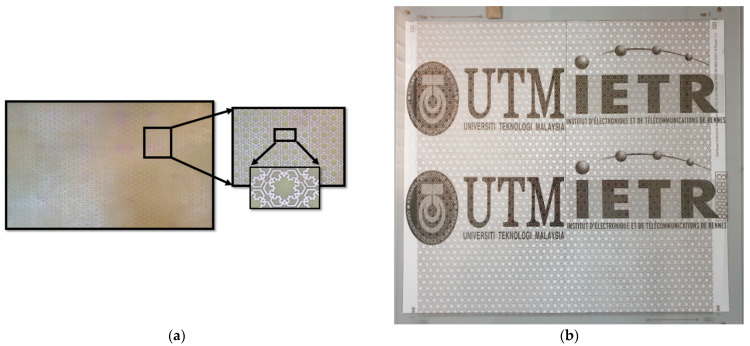
The fabricated prototype of MFSS on both substrates; (**a**) FR4 substrate (210 × 297 mm^2^) and (**b**) polycarbonate substrate (300 × 300 mm^2^).

To evaluate the performance of the fabricated MFSS prototype, we characterised the prototype experimentally. The FSS 3 and the opaque MFSS was fabricated using the FR4 substrate through the LPKF Protolaser U4 laser machine, in which was performed at IETR (Université de Rennes 1, Rennes, France) (Figure 13a). The opaque MFSS unit cell with identical parameters was fabricated on the polycarbonate substrate for optical transparent application using the screen-printing technique that was performed at SERIBASE Industrie (Chateau-Gontier, France) (Figure 13b).

**Figure 14:**
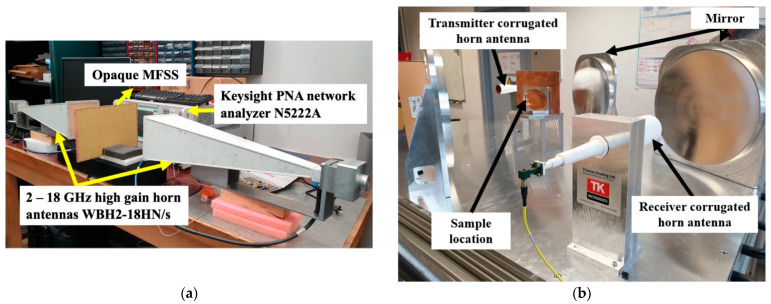
The free space measurement setups for frequency range of (**a**) 2 GHz to 18 GHz, and (**b**) 18 GHz to 40 GHz.

The prototype free space measurement was performed across 3 different microwave spectral bands (2 GHz–18 GHz, 18 GHz–26 GHz and 26 GHz–40 GHz). Figure 14a shows the first measurement setup that is performed by using the Keysight PNA Network Analyzer N5222A, that connected with a pair of the 2–18 GHz high gain horn antennas WBH2-18HN/s (transmitter and receiver), which was focused on the measurement of frequency range from 2 GHz until 18 GHz. The second measurement setup utilized the Quasi-optical measurement setup (a vector network analyzer, a pair of the corrugated horn antennas (transmitter and receiver), two pairs of the successive reflective mirrors) by TK Instruments for the measurement of frequency range from 18 GHz to 40 GHz, as depicted in Figure 14b. The transmitting and receiving horn antennas were placed in a parallel orientation to measure $T_{xx}$, whereas $T_{yx}$ was measured in orthogonal orientation.

**Figure 15:**
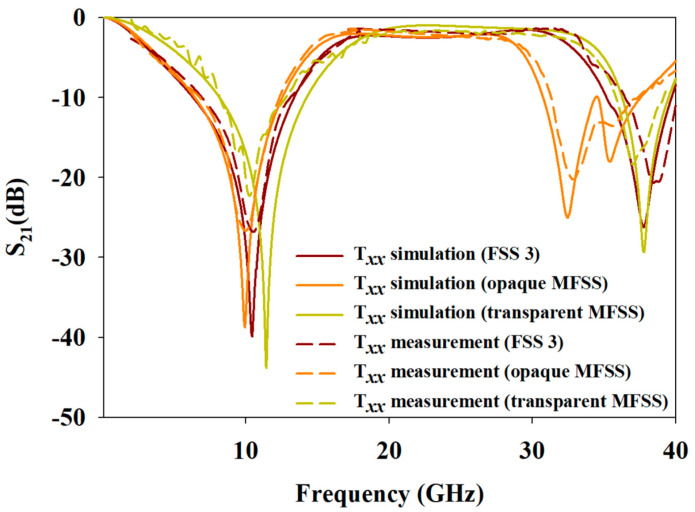
Experimental $T_{xx}$ versus simulation $T_{xx}$ of FSS 3, opaque MFSS, and transparent MFSS.

To ensure brevity, only $T_{xx}$ and $T_{yx}$ performance validations of FSS 3, opaque, and transparent MFSS were considered in this paper. Based on Figure 15, the resonant frequencies of the simulated and measured $T_{xx}$ were in good correlation with FSS 3, opaque MFSS, and transparent MFSS. However, a slight shift in $f_{r}$ between the simulated and measured $S_{21}$ stemmed from multiple reflections from the ambient surrounding as the measurement process was conducted in free space surrounding.

**Figure 16:**
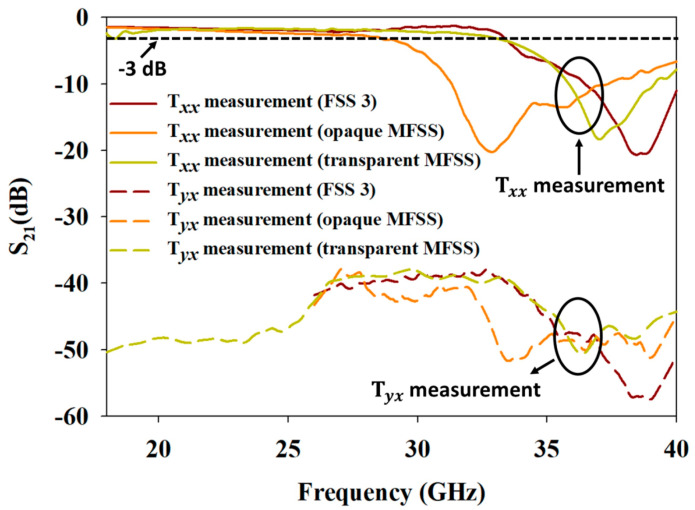
Experimental $T_{yx}$ of FSS 3, opaque MFSS, and transparent MFSS.

Figure 16 illustrates the measured $T_{yx}$ across 18-40 GHz microwave spectral bands, as the bandpass frequency response of FSS 3, opaque MFSS, and transparent MFSS, within the frequency region. Referring to Figure 16, the $T_{yx}$ level, especially within the bandpass frequency range, was measured at −37 dB and below.

To date, the investigation of FSS with low $T_{yx}$ has been rarely documented, except for the application of quasi-optical diplexer, as well as multichannel millimetre- and sub-millimetre-wave radiometers (Earth observation missions). Low $T_{yx}$ level of FSS was achieved at 21 dB (nested annular slot element) and 25 dB (two nested short circuited loop slots and a comparatively wide dipole slot element), respectively, within narrow frequency range of the sub-millimetre wave [53,54]. Meanwhile, [55] reported a lower $T_{yx}$ level of 30 dB across the wider bandpass responses of Ku and Ka bands with FBW of 44%, by using a double square loop element with double layer substrate configuration, in comparison to those reported in [53] and [54]. However, the double layer substrate configuration led to thicker FSS, which could cause difficulty during the fabrication process. Moreover, the $T_{yx}$ yielded by the previously proposed FSS was only available within a narrow band of frequency range and not wideband (FBW ≥ 50%). Turning to this present study, the proposed MFSS recorded further lower $T_{yx}$ of −37 dB within a wideband bandpass frequency response of 59.25% by using only single layer substrate configuration. The $T_{yx}$ level was maintained within the wideband frequency range with FBW of 62.78% when MFSS was used for optical transparency application.

## 5. Comparative Study

The comparison of the opaque and transparent MFSS with previous works is shown in Table 2. The wideband response (FBW > 50%) was only achievable by using multilayer FSS. However, the innovation of FSS element enabled a wideband response to be achieved by using a single layer FSS with one layer metallic conductor [8,24,25,26,27]. Although the available FSSs offer wider wideband bandstop frequency response (FBW > 80%) when compared to the proposed opaque MFSS (64.35%) and transparent MFSS (54.22%), the FSS structural element could not be applied for optical transparency application; see [8,24,25,26,27]. This is because the trace width of the metallic conductor of the previously proposed FSS structural element exceeded 0.8 mm, in which the metallic conductor occupied a wide area of the FSS unit cell and decreased the optical transparency of the FSS. On the other hand, for FSS with a trace width lower than 0.8 mm, only narrow bandstop response (FBW < 30%) with high angular and polarised stability was attained; see [56,57].

As for the bandpass frequency response, both opaque and transparent MFSS recorded higher FBW of 59.25% and 62.78%, respectively, when compared to the FSS proposed in [58] that recorded only 50% bandpass response. In comparison with transparent MFSS proposed in past studies (see [28,29,30,31,32,33]), the transparent MFSS proposed in this present study displayed the advantage of having wideband bandstop and bandpass responses, as well as the ability to provide high optical transparency above 70.3%, but angular stability limited to only 25°.

## 6. Conclusions

This study has proposed an optically transparent MFSS wideband response with low cross polarisation. Initially, the assessed opaque MFSS generated wideband frequency response with low cross polarisation. With a similar MFSS structural element, the MFSS was further utilised for optical transparency application and yielded wideband bandstop, bandpass frequency responses, and high reduction of cross polarisation lower than −37 dB, especially at bandpass frequency response with transparency level exceeding 70%. The simulation and measurement performances revealed good yield for both the opaque and transparent MFSS. The transparent MFSS appeared to be highly essential in providing a soft visual impact, especially for smart city and vehicle window applications.

## Figures and Tables

**Table 1:** The optical transparency of the evolution of the MFSS unit cell.

Type of Elements	Total Area of the Unit Cell (mm^2^)	Area of the Metal Element (mm^2^)	Optical Transparency (%)
FSS 1	42.55	36.27	5.09
FSS 2		26.19	38.4
FSS 3		22.15	42.0
MFSS		12.62	70.3

**Table 2:** The comparison of the opaque and transparent MFSS with previous works.

Ref.	Layers	Total Thickness	Size Dimension	Angle Stability	*T* (%)	Operating Frequency (GHz)	FBW
[8]	1S 1C	0.03 λ	-	30°	-	8.02–18.20	80.28% (−10 dB Bandstop)
[58]	1S 1C	0.02 λ	0.25 λ	45°	-	8.00–13.00	50.00% (−3 dB Bandpass)
[24]	1S 1C	0.04 λ	0.20 λ	80°	-	2.5–13.23	136.00% (−10 dB Bandstop)
[25]	1S 1C	0.03 λ	0.30 λ	30°	-	4.85–17.23	112.14% (−10 dB Bandstop)
[26]	1S 1C	0.01 λ	0.11 λ	60°	-	3.05–10.73	111.47% (−3 dB Bandstop)
[27]	1S 1C	0.04 λ	0.13 λ	80°	-	3.10–10.80	110.79% (−10 dB Bandstop)
[56]	1S 1C	0.05 λ	0.12 λ	80°	-	~5.75–7.75	~29.60% (−10 dB Bandstop)
[57]	1S 1C	0.02 λ	0.16 λ	85°	-	~5.00–6.75	~29.79% (−10 dB Bandstop)
[28]	1S 1C	0.004 λ	0.25 λ	45°	Semi–transparent	~9.00–11.30	~22.78% (−10 dB Bandstop)
[29]	1S 1C	0.0002 λ	0.11 λ	60°	Semi–transparent	1.50–2.50	50.00% (−10 dB Bandstop)
[30]	1S 1C	0.009 λ	0.31 λ	90°	transparent	~11.00–14.50	~27.00% (−10 dB Bandstop)
[31]	1S 1C	0.032 λ	0.32 λ	60°	≥95%	*f_r_* = 2.40	≤1.00% (−20 dB Bandstop)
[32]	1S 1C	0.007 λ	0.15 λ	30°	76.2%	*f_r_* = 1.80, 3.00 and 4.90	Very narrow BW (−10 dB Bandstop)
[33]	1S 1C	0.2 λ	0.68 λ	-	Radioscreen + acrylic = 50%VeilSheild + acrylic = 70%	27.00–35.00	25.81% (−10 dB Bandstop)
This work (Opaque MFSS)	1S 1C	0.05 λ	0.23 λ	25°	-	6.43–12.53	64.35% (−10 dB Bandstop)
						15.49–28.53	59.25% (−3 dB Bandpass)
						30.74–37.55	19.94% (−10 dB Bandstop)
This work (Transparent MFSS)	1S 1C	0.06 λ	0.27 λ	25°	70.3%	8.16–14.23	54.22% (−10 dB Bandstop)
						17.67–33.84	62.78% (−3 dB Bandpass)
						36.27–39.50	8.53% (−10 dB Bandstop)

## Data Availability

Data sharing is not applicable to this article.
